# Cell Mechanosensitivity: Mechanical Properties and Interaction with Gravitational Field

**DOI:** 10.1155/2013/598461

**Published:** 2012-12-26

**Authors:** I. V. Ogneva

**Affiliations:** State Research Center of Russian Federation Institute of Biomedical Problems, Russian Academy of Sciences, 76-a, Khoroshevskoyoe shosse, Moscow 123007, Russia

## Abstract

This paper addressed the possible mechanisms of primary reception of a mechanical stimulus by different cells. Data concerning the stiffness of muscle and nonmuscle cells as measured by atomic force microscopy are provided. The changes in the mechanical properties of cells that occur under changed external mechanical tension are presented, and the initial stages of mechanical signal transduction are considered. The possible mechanism of perception of different external mechanical signals by cells is suggested.

## 1. Introduction

The appearance of life on Earth and the evolution of all living organisms occurred under the influence of external physical fields, gravity, and electromagnetic fields. The formation of a cell—the basic building unit of life, which is capable of an independent existence under these physical conditions meant that the cell's physical properties had to have been such that they enabled it to exist under the influence of these physical fields.

The most constant external physical field is, certainly, the gravitational field. Since the cell is being formed under the influence of an external mechanical field, its mechanical properties, on the one hand, should be such that they enable it to function under the conditions of this field. On the other, hand however, the cell should also be capable of responding to changes in the external mechanical conditions and adapt to them, while not forgoing its ability to reproduce and maintain itself.

Any mechanical system, for example the cell, in the external field is in tension (from a mechanical point of view), and as such the cell forms its structure and internal mechanical tension in accordance with the vector and amplitude of this external force. A change in the external force (either its vector or amplitude) will naturally cause a change in the mechanical tension of the cell and lead to its deformation. The level of significance, and consequences, of these deformations on the essential activities of the cell will depend on the cell's mechanical properties and the sensitivity of its mechanosensors.

Nevertheless, all cells can be divided into two types: cells that form internal tension only in response to an external force, and cells that are also able to generate their own mechanical force, for example, muscle cells. Muscle cells have a specific structure, including a well-developed cytoskeleton that takes up the larger part of the cell and which forms the contractile apparatus. This submembrane (cortical) cytoskeleton of muscle cells is generally similar to the cortical cytoskeleton of nonmuscle cells, except for in several special points, that is in the projection of Z-disc and M-line on the membrane.

Therefore, the problem of cellular mechanosensitivity can be posed as a set of questions: what are different cells' mechanical properties; what is the magnitude of force capable of causing a cellular response; what are the changes in the cellular mechanical and biochemical properties under the changed external mechanical conditions, and finally, what is the cell mechanosensor and how the cellular response is achieved?

## 2. Mechanical Properties of Cells

### 2.1. Cells Capable of Generating a Mechanical Force-Cardiomyocytes and Skeletal Muscle Fibres

With the advent of atomic force microscopy, experimental studies on the mechanical properties of different cells were intensified [1]. Primarily, the focus was on muscle cells, in one of the first studies the stiffness of the muscle fibres was compared with the stiffness of human umbilical vein endothelial cells.

Evidently, the initial interest in muscle cells was connected with the fact that these cells specialize in generating mechanical force and that their mechanical properties determine the force they can produce.

Consequently, Mathur et al. [2] generated one of the first datasets concerning the mechanical properties of intact muscle fibres of the skeletal muscle and myocardium, in comparison with endothelial cells, by using liquid-based atomic force microscopy. The main working hypothesis was that the Young's modulus and viscosity would differ in these three types of cells because of their different structures and functional roles. The experimental samples used were fibres of the rabbit myocardium, C2C12 myoblasts from C3H adult mice and human umbilical vein endothelial cells (HUVEC). The authors showed that the Young's modulus of the endothelial cells was *E* = 6.8 ± 0.4 kPa in the area of nucleus, *E* = 3.3 ± 0.2 kPa on the cell body, and *E* = 1.4 ± 0.1 kPa at the cell edge. As opposed to the endothelium, systematic changes in the Young's modulus of the skeletal muscle fibres and cardiomyocytes, based on the location of the cantilever contact point on the surface, could not be found. For the cells of the myocardium, the Young's modulus was = 100.3 ± 10.7 kPa, and for the cells of skeletal muscles *E* = 24.7 ± 3.5 kPa. Thus, the fibres of the myocardium are the stiffest, and consequently more stable to deformation, which in author's opinion, could be explained by their constant rhythmic activity.

A group of researchers headed by Defranchi et al. [3] carried out experiments to study the structure and transversal stiffness of the sarcolemmas of fully differentiated fibres under different conditions. The experiments were carried out on muscle fibres of the skeletal muscles of CD1 mice. Measurements of the mechanical properties were made in contact mode, the maximum applied force to the fibre membrane was 1 nN, and indentation depth varied from several hundreds to thousands of nm. The value of the Young's modulus presented in the study was *E* = 61 ± 5 kPa.

Thus the values of the Young's modulus found by Defranchi et al. exceeded, by approximately 2.5 times, the results provided by Mathur et al. This is, probably, due to Defranchi et al. using fully differentiated cells in their studies, while Mathur et al. used myoblasts. Such differences in the mechanical properties of muscle fibres at different stages of differentiation can show that the ontogenetic dynamics of protein expression, which forms the structural base of the transversal stiffness, is uneven. The first study addressing this question was conducted by Collinsworth et al. [4]. They studied the mechanical properties of muscle cells at different stages, from myocytes to muscle fibres, and found a significant increase in the Young's modulus on the eighth day after the start of differentiation. Thus, for undifferentiated myoblasts the Young's modulus was *E* = 11.5 ± 1.3 kPa, while on the 8–10th day of differentiation, it was *E* = 45.3 ± 4.0 kPa. At that viscosity that was assessed by hysteresis formed at direct and reversal route of cantilever during registration of force curves was not changed during differentiation. The author's hypothesis concerning the connection between the changes in Young's modulus and the formation of tubulin microtubules was not confirmed experimentally, as the Young modulus and viscosity of muscle cells were not changed after processing with colchicines (in concentrations of 0.4 *μ*g/mL for 2 hours) or taxol (10 *μ*M for 2 hours). However, processing with cytochalasin D (in concentrations of 3 *μ*M for 5–30 minutes) or blebbistatin (in concentrations of 50 mM for 5–30 minutes) did cause a significant decrease in Young's modulus without an accompanying change in viscosity properties. Because of this, the authors connected changes in stiffness properties of the muscle cells during differentiation with the development of an actin-myosin system. It is important to note that Collinsworth et al. did not analyse the contribution of the nonsarcomere cytoskeletal proteins in the transversal stiffness of muscle fibres, even though processing by cytochalasin D can cause damage of actin filaments of the cortical layer.

We developed a method that enabled us to define the transversal stiffness of different parts of the muscle fibre [5]. Consequently, for different parts of rat fibre's membrane in the relaxed state, the following changes in transversal stiffness were seen: membrane at the projection of the Z-disc—3.08 ± 0.14 pN/nm (m. soleus), 2.24 ± 0.18 pN/nm (m. medial gastrocnemius), 2.98 ± 0.29 pN/nm (m. tibialis anterior), 16.0 ± 1.3 pN/nm (heart); membrane at projection of the M-line—1.98 ± 0.07 pN/nm (m. soleus), 1.53 ± 0.07 pN/nm (m. medial gastrocnemius), 1.43 ± 0.26 pN/nm (m. tibialis anterior), 9.9 ± 0.6 pN/nm (heart); membrane in the area of the semisarcomere—3.05 ± 0.03 pN/nm (m. soleus), 2.87 ± 0.12 pN/nm (m. medial gastrocnemius), 2.98 ± 0.29 pN/nm (m. tibialis anterior), 7.1 ± 0.4 pN/nm (heart) [6–8].

Conducted measurements of the fibres of different skeletal muscles and cardiomyocytes show that the transversal stiffness of myocardial fibre membranes, in all parts, are definitively higher than the transversal stiffness of the membrane of skeletal muscle fibres. The increases in transversal stiffness of the muscle cell membranes are connected to the development of the cytoskeleton, in particular at cell maturation. Evidently, a more developed cortical cytoskeleton can explain the increase in cardiomyocyte stiffness, while with the fibres of skeletal muscles this increase is likely explained by high mechanical load.

### 2.2. Nonmuscle Cells

Costa et al. [9] studied the mechanical properties of human aorta endothelial cells (HAEC). The measurements were conducted in contact mode in liquid, indentation depth was 200 nm. The authors found that there were two types of cells which differed in Young's modulus: one type had a Young's modulus of 5.6 ± 3.5 kPa, while others had one of 1.5 ± 0.76 kPa. However, after processing with cytochalasin B (in concentrations of 4 *μ*M), there were no differences in their mechanical properties while their Young's modulus corresponded to the data of Mathur et al. [2], who also studied endothelial cells but only from the umbilical vein and not from the human aorta. Moreover authors showed that in this case the cells' mechanical properties were defined by an actin cytoskeleton.

A similar approach was used by Martens and Radmacher [10] with the aim of demonstrating the main contributions of the actin cytoskeleton in the stiffness of human fibroblasts, which was assessed before and after addition of blebbistatin and the Rho-kinase inhibitor Y27632. The authors showed that, at addition of blebbistatin, the Young's modulus decreases from 20 kPa to 8 kPa over the course of 30–60 minutes, while processing with Y27632 did not cause any significant mechanical effects. In the authors' opinions therefore, these results prove that the tension caused by myosin determines the cell stiffness.

Cai et al. [11] showed that measurements of a cell's mechanical properties can be used as a diagnostic parameter, for example in the analysis of lymphocyte degeneration. Normal human lymphocytes and human T-lymphoblastic Jurkat cells were examined. Atomic force microscopic images showed that the cell profiles, that is, surface striations, are similar in both types. However, although the stiffness of normal lymphocytes is 2.28 ± 0.49 mN/m, for Jurkat lymphocytes it is 4.32 ± 0.3 mN/m.

In researching pathological forms of erythrocytes in patients with hereditary spherocytosis deficit of glucose-6-dehydrogenase, thalassemia and with anisocytosis, Dulińska et al. [12] showed that their stiffness was increased in comparison to normal cells. Lekka et al. [13] meanwhile assessed erythrocytes in patients with confirmed diagnoses of coronary disease, hypertension, and diabetes mellitus, and compared these with erythrocytes of healthy volunteers. The authors showed that the mean Young's modulus values and the width distribution of its values were markedly higher in patients with diabetes mellitus, and in smokers, in comparison to healthy people. Moreover, the Young's modulus of erythrocytes increased with the age of patients.

According to the patient's age, stiffness of chondrocytes also changes, but in this case it was a decrease that was seen. Hsieh et al. [14] showed that the stiffness of chondrocytes in young men is 0.096 ± 0.009 N/m, and in patients with osteoarthritis (old age), it is 0.035 ± 0.005 N/m.

Except those mentioned above, the most widely studied cells are those of the human bone marrow, which are represented in particular by mesenchymal stem cells (hMSCs) and osteoblasts (hOBs). Docheva et al. [15] studied the topography and mechanical properties of two types of hMSCs: rapidly renewing cells (RS) and flat cells (FC), also in addition to hOBs and cells of the osteosarcoma line MG63. Atomic force microscopy images showed that FC and hOB surfaces are strongly striated, while in RS and MG63 they are comparatively smooth. Moreover RS and MG63 cells were flatter on the fibrous substrates than on smoothed surfaces on the slide. In contrast, in cells with greater surface area, that is, FC and hOBs, flatness was not dependent on the substrate and was more evident in comparison with RS and MG63 cells. The authors tried to explain this result as being due to different degrees of cell adhesion, taking into account the higher content of focal adhesion complexes in FC and hOBs than in RS and MG63 cells. When examining the mechanical properties of these types of cell, Docheva et al. [15] showed that the Young modulus in normal cells, that is, RS, FC, and hOBs, have comparable values across the three different substrates while in MG63 cells, it significantly increases depending on the collagen of the I type.

Takai et al. [16] also studied the dependence of mechanical properties on the substrate. The objects of their study were MC3T3-E1 osteoblast-like cells, tested on the following different substrates: fibronectin (FN), vitronectin (VN), type-I collagen (COL I), fetal bovine serum (FBS), poly-L-lysine (PLL), and no substrate, that is, tested directly on the slide. The results obtained by the authors showed that the Young's modulus of osteoblasts that were bound to extracellular matrix proteins (FN, VN, COL I, FBS) through integrins, was higher than in similar cells on PLL, or on the slide alone where interactions are nonspecific. Also, formation of F-actin stress-fibrils was observed on FN, VN, COL I, and FBS, while there were only very small quantity fibrils of F-actin found for PLL or on the slide alone. Damage to the actin cytoskeleton decreases the Young's modulus of osteoblasts on FN so that it reaches the level of that for cells on the slide alone. At the time damage of microtubules did not cause any significant effects. Takai et al. [16] suggest that increases in the Young's modulus of osteoblasts on FN occurred because of rebuilding of the actin cytoskeleton in response to interactions with extracellular matrix proteins.

A more detailed study of the influence of the extracellular matrix on the morphology and mechanical properties of cells was conducted by Yim et al. [17]. Here, the authors suggested that nanotopography of the extracellular matrix could influence cell behaviour by changing the interaction with integrins and/or focal adhesion complexes. hMSCs cultured on 350 nm layers formed by stiff TCPS (tissue-culture polystyrene) or soft PDMS (polydimethylsiloxane) were selected as the object of this study. Study results showed that in cells cultured on both the TCPS and PDMS, there was a decrease in expression of some integrin subunits (*α*2, *α*6, *α*V, *β*2, *β*3, *β*4) and a flattening of the actin cytoskeleton occurred when compared with a control, where a hard actin reticulum with a random sequence distribution was observed. Moreover, on the stiff TCPS, the Young's modulus of hMSCs was lower than on the soft PDMS. Furthermore, nanotopography effects did not affect the mechanical properties, although in cells cultured on the PDMS, the value of the Young's modulus was notably lower than in cells cultured on TCPS. From results obtained by Yim et al. [17], it can be concluded that both the nanotopography and substrate stiffness have an influence on the cell mechanical properties, but that the role of nanotopography is dominant in the regulation of the cytoskeleton state.

Thus, the data of different authors enables us to suggest that the stiffness of nonmuscle cells depends significantly on the type of substrate on which they are cultivated. In addition, the nanotopography of the substrate has a substantial significance that can be connected to the formation of adhesion sites and development of a submembranous cytoskeleton. Data concerning the increased stiffness of proliferated cells, which are also characterised by an increase in protein content forming cortical cytoskeleton, also demonstrates this. 

## 3. The Magnitude of Force Capable of Causing Cellular Responses

The process of transformation of physical signals into biochemical ones, and the formation of appropriate cellular responses, is called mechanotransduction or mechanosensitivity [18].

Understanding of the molecular basis of mechanotransduction is impossible without knowledge of the amplitude and distribution of forces influencing cells. However data presented in the literature and conducted in this direction are very limited. First Davies et al. [19] suggested that the cellular response to an applied external mechanical force could be mediated by its transfer to the cell nucleus and also through intercellular interactions and adhesive contacts. The central idea of these authors is that the force affects more structures, although it becomes lessened with increasing distance from the point of force application. Nevertheless, this is not always the case as these authors showed in additional work using intermediate filaments as a marker for changing forces [20, 21]. Similar experimental approaches were used by several other groups whose purpose was the mapping of stresses that appeared in the cell in response to the applied external force [22–24]. All these results prove that forces have a complex, heterogeneous nature that is mediated by several proteins and their complexes.

Thus, a key question is what is the magnitude force that is able to cause a cellular response?

Force can be exerted on the cell by different experimental methods, and if the applied force is sufficient, the cellular response can be analysed. In this way, Huang et al. [18] showed that a shear stress equal to 1 Pa is critical to endothelial cells. This force is approximately 1 nN when recalculated for an area of 1 *μ*m^2^. If this force is balanced by response of focal-adhesive complex only taken over 1% of the whole square but the load on it increases by 100 times. It is relatively difficult to compare these values with the force measured in experiments with alternative deformation because there are no measurements of the force of cell interaction and elasticity of the substrate. However, by taking into account that the Young's modulus is 1 kPa, a force of 100 Pa will cause a relative deformation equal to 10%.

The analysis of the force of cell interaction and the substrate without overlapping of information can be the alternative for such measurements. The point is that by knowing the Young's modulus of the substrate, it is possible to measure its deformation while putting the cell able to bind on it. While studying fibroblasts, Balaban et al. [25] showed that the force of interaction is 5.5 nN/*μ*m^2^. Assuming the close packaging of integrins in the focal adhesion complex, the authors assessed the force necessary to cause changes in the conformation of every integrin to be at the level of several pN.

The level of force necessary for a change of conformation in other proteins also can be determined. The force necessary to break the link between two proteins can be taken as the upper assessment. So for the dissociation of fibronectin from integrin, a force of 30–100 pN is required [26]. Meanwhile, a force of 3–5 pN is necessary for the unfolding of fibronectin domains [27]. Ferrer et al. [28] showed that the force required for alpha-actinin to dissociate from actin, and also for filamin, is 40–80 pN.

However, these conformational changes can be significant if they exceed the level of heat noise; *kT* is approximately 4 pN·nm, so that when comparing with the characteristic deformations on the level of 1–10 nm, the force should be not less than 4 pN. This is comparable to the magnitude of force generated by one myosin head at muscle contraction [29], which increases the assurance of the accuracy of the assessments made by Huang et al. [18].

## 4. Potential Mechanosensors

The extracellular matrix and membrane proteins, mechanosensitive and/or other ion channels, structures of the submembrane (cortical) cytoskeleton, and intracellular structures, in particular, could all act as mechanosensors.

### 4.1. Extracellular Matrix and Membrane Proteins

It has been shown that the applying of stretching force to a culture of neurons or smooth muscle cells, through the extracellular matrix leads to an increase in the polymerisation of microtubules [30, 31]. Integrins, which form connections with various proteins in the extracellular matrix, such as fibronectin and vitronectin, form a primary site of transduction and consequently, can be considered as mechanosensors. From the intracellular side, a number of proteins are concentrated near the focal adhesion complex that directly interact with *α*- or *β*-subunits of the integrin heterodimer. These proteins include paxillin, focal adhesion kinase, and caveolin, where paxillin and focal adhesion kinase can connect to a large number of other proteins, thus forming signalling cascades. Besides these, tensin, alpha-actinin, and filamin can also connect with integrins and the cortical cytoskeleton, as they have the appropriate domains, and work in conjunction with integrins and actin [32]. Furthermore, alpha-actinin has more than one domain with which it interacts with actin and form an actin network. The structure of the focal adhesion complex is characterised by large number of proteins, all located in the immediate vicinity of each other, and so this feature complicates the analysis of the contribution of each in mechanotransduction and does not allow for determinations of which have the more dominant roles. However, it is obvious that an external mechanical force can lead to conformational changes in one or several of the proteins of the focal adhesion complex, further triggering the cascade of underlying signalling pathways.

### 4.2. Mechanosensitive Ion Channels

Mechanical stretching of cellular membranes, for example using the patch-clamp technique, changes the transportation activity of mechanosensitive ion channels as a result of conformational changes or changes in the lipid bilayer [33, 34], or in the gate domains of the channel itself [35, 36]. In addition, the majority of channels studied respond to cellular stretching, but not to compression.

Prokaryotic mechanosensitive ion channels have been described in many experimental studies and reviews. One of the most well-characterised mechanosensitive channels is the bacterial MscL, which has a pore with a large diameter and low ion selectivity. This channel possesses the highest conductivity known, about 10^3^ pCm [37], and can be regulated by membrane tension, as shown in experiments using the patch-clamp technique. Increasing the membrane tension, supervised by variation of depth of absorption to a pipette, caused an increase in the conductivity of channels when the forces operating on the channels exceed a certain size [37]. The authors showed that the tension in this case was equal to 10^−2^ Pa·m, that is slightly lower, than tension, leading to gap (6 × 10^−2^ Pa·m) that can have a big physiological value, for example, in the swelling of a bacterial cell that occurs as a consequence of osmotic shock. The results of molecular dynamic modeling [38], based on the data on MscL crystal structure, show that such changes in membrane tension lead to the formation of pores with a diameter of 0.5 nm [39]. At the same time, experiments *in vitro* showed that the diameter of an open pore was 3-4 nm [40]. However, the adequacy of the results of the experimental situations *in vivo* is under question.

Few eukaryotic channels have been identified as mechanosensitive channels: TRP channels, K(2P) channels, MscS-like proteins, and DEG/ENaC channels [41]. The transient receptor potential (TRP) protein superfamily consists of a diverse group of cation channels that have important roles in cells of the nervous system and in nonexcitable cells [42]. It has been shown that the underlying cytoskeleton and scaffolding proteins can influence the regulation of gating in TRP channels [43].

The two-pore-domain K(+) channels, or K(2P) channels, have four transmembrane regions, act as dimers, and are widespread in different tissues (in both excitable and nonexcitable cells). They are thought to play a major role in setting the resting membrane potential of many cell types. K(2P) channels are quasi-instantaneous and noninactivating, and they are active at all membrane potentials and insensitive to the classic K(+) channel blockers. The TWIK-related (TREK)-1 and TWIK-related arachidonic acid-stimulated K(+) (TRAAK) channels were the first cloned polyunsaturated fatty acid-activated and mechanogated K(+) channels [44].

Epithelial sodium channels (ENaCs) are a subfamily of ion channels within the degenerin/ENaC (DEG/ENaC) superfamily. These ion channels are found in different sodium-absorbing epithelia, including the epithelium of the colon, lung, and distal nephron; their activity represents the rate-limiting step for sodium uptake, and thus transepithelial water movement [45]. There is growing evidence concerning the activation of ENaC by mechanical forces, and at least laminar shear stress seems to be an adequate stimulus of physiological significance [46, 47]. Highly selective epithelial Na^+^ channels are expressed in various vertebrate epithelia where they are exposed to shear forces such as the distal nephron [46, 48], airway epithelia [49], vascular tissue [50–52], and sensory nerve endings, indicating participation in mechanosensitive processes [53].

### 4.3. Cortical Cytoskeleton

Today the role of cortical cytoskeleton in the regulation of ion channels is quite well established. It has been shown that condensation of cortical actin under the plasma membrane occurs as a result of the phosphatase inhibitor, calyculin A, suppressing a depot-dependent input of calcium in smooth muscle cells in culture [54], and also cytochalasin D [55]. With the use of patch-clamp techniques, it was shown that actin microfilaments take part in the regulation of chloride channels [33, 56], Na+-K+-ATPase [57], electroexcitable sodium channels in brain cells [58], and sodium channels in cells of reabsorbable epithelium [59]. Dismantling of actin filaments by cytochalasin D leads to the activation of sodium channels in K562 cell lines, while actin polymerisation on the cytoplasmic side of an external membrane of a cell causes channel inactivation [60]. Thus, the fragmentation of actin filaments, as associated with a plasma membrane, caused by cytosolic actin-connecting Ca-sensitive proteins that are similar to endogenous gelsolin, can be a major factor in inducing the activity of sodium channels in response to increasing intracellular concentrations of calcium ions in K562 cell lines [61, 62].

In addition to the examples mentioned above, there are data which indicate that an association with the lipid microdomains of cholesterol-rich plasma membranes (rafts) could be the essential factor in defining the activity of integrated membrane proteins, including ionic channels [63–68]. The disruption of the membrane structure and raft integrity, caused by a decrease in the level of membrane cholesterol, interferes with execution of cellular functions, including reorganisation of the actin network [66, 69]. Therefore, it was shown that a partial extraction of membrane cholesterol using methyl-beta cyclodextrin, at concentrations of 2.5 or 5 mM, inhibited mechanosensitive activation of channels in K562 cell lines [70, 71]. In cells with a lowered cholesterol content, there was an observed increase in the threshold of activation, and a decrease in the probability of channels being in an open state. Thus, measurements of mechanosensitive flows in various conditions, and complementary data from fluorescent microscopy, indicate that suppression of activity of mechanosensitive channels is mediated by the reorganisation of actin, which is initiated, according to the authors, by disruption of raft integrity due to decreases in the levels of membrane cholesterol [70, 71].

Considering that the initiation of many intracellular signalling pathways is a result of membrane-bound proteins that amplify, at increased speed, the lateral diffusion of a signal, Jalali et al. [72] offered another theory of mechanotransduction. Using fluorescent labels, they estimated the speed of their migration in phospholipids bilayer, upon applying a shearing stress, and the dependence on how this stress was increased, that is, smoothly or in steps. In the case of a step-wise increase, the diffusion factor increased, while in case of gradual increase of stress this decreased. In their review, Huang et al. [18] assume, being guided by data from Butler et al. [73], that the effect of increases in the lateral diffusion is connected with ERK and JNK activation. Nevertheless, it still remains unclear how shearing stress leads to changes in membrane fluidity.

### 4.4. Intracellular Structures

It is well known that the action of external forces can lead to changes in the levels of gene expression. In combination of facts that the forces enclosed through the membrane-connected receptors, in certain cases, can lead to nucleus deformations [74], it is possible to assume a direct influence of external forces on chromatin and, as a result, on expression level [18]. Forces in this case could be transduced through the cytoskeletal network to the nuclear envelope, and then, through the laminin network to chromatin.

In addition, the action of external forces could be transduced to microtubules, leading to their disruption, depolymerisation, and the initiation of a signalling pathway [75].

It should be noted that conformational changes in various proteins could potentially be applied to a mechanosensory role, but there is no direct, practical proof of this. However, there is at least one example that a biochemical response can be caused by conformational changes of proteins. As discussed above, the folded domains of fibronectin can be exposed through the application of a stretching force to the molecule, which leads to fibril formation. This process was investigated both experimentally and through molecular-dynamic modelling methods [76, 77] and, as result, it was shown that a force of 3–5 pN is sufficient for the unfolding of domains, and that a subsequent force of 5 pN can lead to a molecule lengthening fivefold in comparison to its initial length [77, 78]. These levels of force are comparable to that, which according to estimates, can initiate mechanotransduction.

Much less is known, however, concerning the various intracellular proteins (e.g., Src-family kinases, vinculin, mDia, and ROCK) which also can be specific “molecular switches,” undergoing conformational changes in response to an external force [79]. In fact, any protein participating in a mechanotransduction from extracellular contacts of a cell can act as a mechanosensor and stimulate the unfolding of both integrin isoforms [80] in addition to the proteins associated with them [81]. Proteins of the focal adhesion complex are also primary candidates for roles as mechanosensors. This becomes particularly obvious in the case of experimental data which shows that the stretching of cells, through the use of detergent (for removal of the cellular membrane), on a pliable substratum can lead to communication strengthening between focal adhesion kinase and paxillin, in the region of focal adhesion [82]. As the cellular membrane was removed in these experiments, the ion channels could not participate in this response.

According to theories proposed by Ingber [83], the cytoskeleton as a whole reacts to changes in mechanical tension through the extracellular matrix, and the associated integrins, leading to the reorganisation of microfilaments and microtubules. In tandem with this, the cortical cytoskeleton, which supports the plasma membrane through the formation of a rigid 3D-framework, is in tension in an external mechanical field [84].

**Figure 1:**
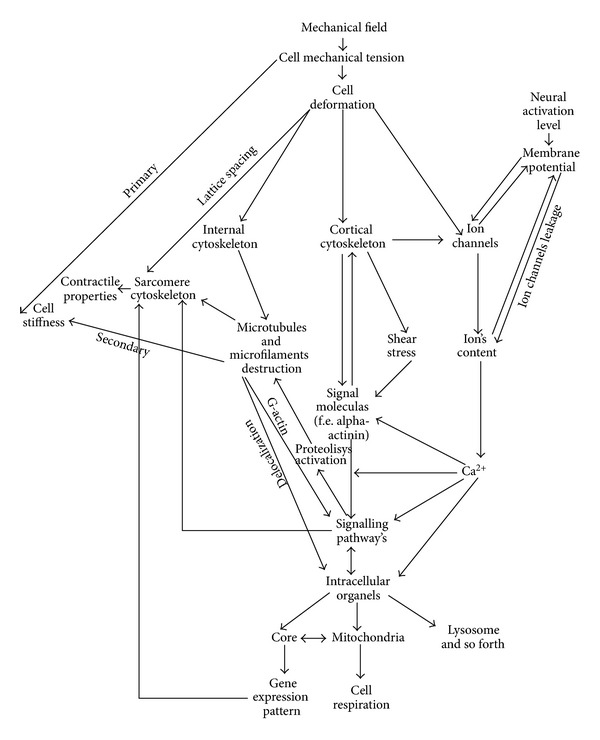
General mechanotransduction schemas. Changes in the external mechanical load cause a change in the internal mechanical tension of the cell and its deformation. Deformations can occur in the ion channels causing changes in their permeability for different ions, for example for calcium, which is a secondary messenger and can activate some signalling pathways. Moreover, deformations can also occur in the cytoskeleton, both in the sarcomere (for muscle cells) and cortical cytoskeleton, causing the release of different signalling molecules and activation of downstream signalling pathways. The final result will be a change in the cell's mechanical properties, its functional activity, and formation of adaptive patterns in gene expression.

Therefore, by summarising the studies above (Figure 1), it is possible to see that practically all possible mechanisms of primary mechanotransduction depend on the condition of the submembrane cortical cytoskeleton structure which determines the mechanical properties of various types of cells, and this is ultimately reflected in stiffness of cells.

Together, a number of experiments that focused on the influence of external mechanical conditions indicated that changes in the vector and modulation of external forces lead to changes in the structural/functional properties of both muscle (specialised for the generation of mechanical tension in cells) and nonmuscle cells.

## 5. Nonmuscle Cell Responses to Changes in External Mechanical Conditions

The changes in cell orientation within a gravitational field can be performed with the help of a horizontal clinostat and RPM—a random position machine [85–90].

Different types of cells have been cultivated under changing gravitational vector conditions. Most of the cells grown under these conditions showed changes in their cell profiles, as well as disorganisation of microtubules and microfilaments, and an increase in the number of apoptotic cells in the culture [91–96], in addition to alterations in mitochondrial localisation and clustering behaviour [56].

The data in the literature show that the influences of gravity vector changes in embryonic stem cells, decreased their ability to form embryoid bodies as a result of clinostation [89, 97]. Conversely, according to data from the same authors, the clinostation of embryoid bodies significantly increased the number of beta-III tubulin-positive cells (early neuroblasts). However, it also gave rise to an insignificant decrease in the number of MAP2-positive cells (late neuroblasts) over the course of spontaneous neuronal differentiation [89, 97].

Mesenchymal stem cells are also sensitive to microgravity conditions; however, there is no common hypothesis in the interpretation of the results obtained from these studies [96, 98–101]. Modelling of microgravity effects, meanwhile, suggested an inhibition of osteogenic differentiation and activation of adipogenic differentiation of mesenchymal stem cells [88, 96, 98–100, 102–104].

According to data cited in the literature, the changes in various cultivated cells can be connected to reorganisation of the actin cytoskeleton [90, 94], particularly, with F-actin destruction, which leads to Rho-dependent signalling pathway activation [98–100], and which can also regulate MAP-kinase cascades as a result of an increase of phosphorylated ERK1/2^MAPK^ levels [96, 98, 103]. The change in differentiation potential of stem cells, after real or modelled microgravity changes, can also be connected to cytoskeleton reorganisation, as some of cytoskeletal structures may take part in the determination of the differentiation pathway [105, 106]. In addition, 24 hours of microgravity modelling caused transient changes in gene expression in mesenchymal stem cells. Some of these genes encoded actin cytoskeleton proteins and the elements associated with this. The modelling also decreased the capability for adhesion in these cells [90].

Thus, the change in the external mechanical (gravitational) vector leads to structural/functional changes in nonmuscle cells. These changes are seen in alterations in division speed and development potential, and are probably connected with cytoskeleton reorganisation. Nevertheless, the primary levels of nonmuscle cellular responses to a change in external mechanical conditions are little studied and so the search for the mechanosensor will not end soon.

## 6. Changes in Original Mechanical Properties of Muscle Cells and Their Cellular Respiration in Response to Changes in External Mechanical Conditions

Results of numerous studies in conditions of real and simulated gravitational unloading testify that negative changes in various bodies and tissues are formed as a result of the action of microgravity.

Skeletal muscles are especially vulnerable to a gravity-free state as the specialised organ that executes position and motor functions. A subject of many studies is the m. soleus for which it has been shown that long exposures to microgravity conditions leads to essential decreases in weight, and changes in fibre atrophy [107–109]. In addition, a decrease in the functionality of both the whole muscle [110, 111] and single fibres [112] also takes place.

Exposure to microgravity also causes various changes in the human cardiovascular system, particularly a cephalic fluid-shift in the cranial direction [113, 114] and a change in the heart's systolic volume [115–117].

Nevertheless, a substantive problem which is obstructing space development by humans, in particular flight to Mars, is the early readaptation period to gravity. Especially important is the reloading of muscle and cardiovascular system functionality, the acceleration of which is impossible without an understanding of the mechanism of development of adaptive changes.

Differently directed changes in the external mechanical conditions of skeletal and cardiac muscles of rats can be achieved by means of a common method; antiorthostatic hindlimb suspension of animals, according to the Ilyin-Novikov method with Morey-Holton modifications [118]. On the one hand, the antiorthostatic suspension of animals leads to hindlimb disuse, while on the other hand, it causes an increase in the mechanical load on cardiomyocytes. In addition, the orientation of skeletal muscle fibres and cardiomyocytes in the gravitational field will change.

According to the scarce data connected with the response of skeletal muscles cells to gravity disuse in the literature, one of the first events to occur, after two days of antiorthostatic disuse of the hindlimbs of mice, is the accumulation of calcium ions in the soleus muscle [119, 120]. Above, we described that the maximal calcium ion accumulation in the soleus muscle fibres of rats and a Mongolian gerbils happens after one day of gravity disuse. For the medial gastrocnemius and tibialis anterior muscles, this maximum was seen a short time later—after seven days of antiorthostatic suspension [121]. The increase in the resting calcium level can lead to calpain activation [122, 123] and the following destruction of the muscle fibre structure. However, the means by which it is accumulated remain unknown. Probably, this effect happens via L-type calcium channels, but the mechanosensitive channels of the TRP family may also take part. In any case, the functioning of the channels incorporated in the membrane depends on the condition of the sarcolemma and the cytoskeleton connected with it. Moreover, we can suppose that the mechanical properties of cardiomyocytes will change, in other ways to that of skeletal muscle fibres, as the load on the cardiac muscle increases in microgravity conditions, while the load on the skeletal muscles of hindlimbs decreases.

### 6.1. Stiffness of Muscle Cells

It is rather difficult to perform a direct evaluation of the native state of the cortical cytoskeleton of muscle fibres. However, the definition of its mechanical properties (transversal stiffness, to be exact) can help in the analysis of its structural changes. In addition, the complicated sarcomere organisation of a muscle fibre enables us to suppose that the transversal stiffness of different parts of the sarcolemma (Z-disk, M-line, and the part between them) will differ from each other. The differentiation of stiffness factors is of great interest, for example, in regarding the signalling role of different proteins of the costamere, which probably depends on its structure.

The methodology based on this above concept enabled us to define the transversal stiffness both of a contractile apparatus and of a membrane with a cortical cytoskeleton [5].

It was shown that the transversal stiffness of different parts of the soleus muscle contractile apparatus in the relaxed, calcium activated, or rigor states decreases, over the course of gravity disuse. It, in practical terms, does not change for the gastrocnemius muscle fibres and increases for tibialis anterior [8, 121]. Meanwhile, the transversal stiffness of the sarcolemma, with a cortical cytoskeleton in the relaxed state, decreases in all the muscles when under antiorthostatic disuse. This happens on the first day of disuse (the soleus muscle fibres decrease from 3.05 ± 0.03 pN/nm to 1.25 ± 0.07 pN/nm; for the tibialis anterior muscle fibres from 3.56 ± 0.28 pN/nm to 2.46 ± 0.13 pN/nm; for medial gastrocnemius fibres from 2.87 ± 0.12 pN/nm to 2.21 ± 0.08 pN/nm), which could be connected with the immediate change in the gravitational vector as an external mechanical factor, thus changing the load on the membrane [8].

Furthermore, for rat cardiomyocytes, it seems, that under the conditions of antiorthostatic disuse, there was an increase in mechanical load early on as a result of hypovolemia. Consequently, the stiffness of the cortical cytoskeleton membrane increased: from 4.03 ± 0.11 pN/nm in the control to 12.3 ± 0.4 pN/nm after 14 days under antiorthostatic disuse [6, 7]. At reloading after antiorthostatic disuse, the stiffness of skeletal muscles fibres increased (transversal stiffness of rat soleus muscle fibres: control—2.94 ± 0.14 pN/nm, after 14-day-display—1.11 ± 0.06 pN/nm, after 3-day reloading 2.92 ± 0.10 pN/nm) [124], while the stiffness of cardiomyocytes decreased (transversal stiffness of rat left ventricle cardiomyocytes: control—4.03 ± 0.11 pN/nm, after 14-day display—12.3 ± 0.4 pN/nm, after 7-day reloading—4.3 ± 0.4 pN/nm) [6, 7], returning to the control level.

### 6.2. Protein Content

Changes in the transversal stiffness factors, both of skeletal muscles fibres and cardiomyocytes, under the conditions of antiorthostatic disuse and the following reloading, correlated with the content of nonmuscle isoforms of actin (that form a cortical cytoskeleton) in the membrane fraction. They were also connected with differently directed changes in nonmuscle isoforms of the alpha-actinin content 1 and 4 of the membrane and cytoplasmic fractions [6, 124].

For soleus muscle fibres, it was shown that the content of beta-actin in the cytoplasmic fraction did not change as a result of 14-day suspension, nor in the following 3-day reloading. However the content of beta-actin in the membrane protein fraction decreased after disuse more than threefold when compared with the control level, but after three days of recovery, it did not differ from the control level, which correlated with the changes in sarcolemma transversal stiffness [124]. Meanwhile, for cardiomyocytes the content of gamma-actin in the cytoplasmic protein fraction also remained at the level of the control during disuse and reloading. However, the content of gamma-actin in the membrane protein fraction significantly increased during the first day of antiorthostatic suspension and continued to rise until the fourteenth day, showing similar dynamics to that of the transversal stiffness change. At the same time, the content of beta-actin in the cytoplasmic and membrane fractions did not change under the conditions of antiorthostatic disuse and the following reloading [6]. It should be noted that the increase of nonmuscle F-actin (beta-actin) content was noticed in cat cardiomyocytes during hypertrophy stimulation [8], although the content of gamma-actin was not defined during this experiment.

The change in the content of nonmuscle actin isoforms, particularly in the membrane fraction, will lead to a structural change in the cortical cytoskeleton as well as changes in actin-binding protein content, particularly alpha-actinin-1 and alpha-actinin-4. These alpha-actinin nonmuscle isoforms can bind calcium ions in micromolar concentrations, whereas calcium concentrations of more than 10^−7^ M fully inhibit alpha-actinin and actin binding [125], as well as lead to the phosphorylation of tyrosine by focal adhesion kinase inside the actin-biding domain [110]. Considering data on the increase in the resting calcium levels of muscle fibres in gravity disuse, interest is drawn towards the calcium-sensitive actin-binding proteins-alpha-actinin-1 and alpha-actinin-4.

For rat cardiomyocytes under conditions of antiorthostatic disuse, the content of alpha-actinin-1 in the cytoplasmic fraction of proteins decreased after seven days of disuse, while in the membrane fraction it increased. In the period of 3-day reloading after 14 days of antiorthostatic disuse, the content of alpha-actinin-1 in the membrane fraction decreased, while it increased in the cytoplasmic fraction. After seven days of recovery, both fractions were the same as the control level [6].

At the same time, in cardiomyocytes, the content of alpha-acitnin-4 in the membrane protein fraction increased during the first day, and starting from the third day its content increased from control levels in the cytoplasmic fraction as well. During the reloading period the content of alpha-acinin-4 in the membrane fraction decreased to the control level. As for the cytoplasmic fraction, it also decreased, but did not reach the control level [6]. In addition, in the rat soleus muscle fibres under conditions of antiorthostatic disuse, the content of alpha-actinin-4 did not change in the cytoplasmic protein fractions. In the membrane fraction, the content of alpha-acinin-4 decreased after 14 days of disuse, and increased after 3-day reloading, although it did not reach the same levels as the control [124].

Very little is known about the role of nonmuscle isoforms of alpha-actinin in skeletal muscles cells or cardiomyocytes. It is known that alpha-acinin-1 is expressed in cardiomyocytes [126] and in skeletal muscles cells, as well as alpha-actinin-4 in different stages of differentiation [127]. Alpha-actinin-1 and alpha-actinin-4 are nonmuscle isoforms of alpha-actinin—a protein that belongs to the spectrin family [128]. They function via antiparallel homodimer binding of actin thread ends [129]. In addition, alpha-actinin-4 connects the actin cytoskeleton to the membrane and facilitates interactions of the cortical cytoskeleton with cytoplasmic signalling proteins [130].

Nevertheless, there is some data, showing that the increase in alpha-actinin-4 content in the cytoplasmic fraction is associated with a decrease of alpha-actinin-1, and with the formation of a cancerous pattern in fibroblasts [131]. There are also results showing that the malignancy of cells, particularly lymphocytes, is followed by an increase of their stiffness, which was measured with the help of atomic force microscopy [11].

Therefore, the increase of cell stiffness is connected with the development of a cortical cytoskeleton, the decrease of alpha-actinin-1 and the increase of alpha-actinin-4 in the protein membrane fraction. Therefore, it can be supposed that the development of a cortical cytoskeleton will lead to the increase in alpha-actinin-1 and alpha-actinin-4 contents in membrane protein fractions.

### 6.3. Cell Respiration

Data obtained by Goffart et al. [127] showed that alpha-actinin-4 can bind to the promoter region of the cytochrome *c* gene, causing an increase in its expression that can influence the efficiency of cellular respiration.

For muscle cells, it was shown that the functions of mitochondria can influence their shape, due to extension or compression of the membrane, that can be mediated by the cytoskeleton [109, 132]. In addition, Milner et al. [133] found that abnormal accumulations of clusters of subsarcolemmal mitochondria, and also swelling of mitochondria with degeneration mitochondrial matrices, in the fibres of m. soleus of null-desmin mice. Moreover, these authors determined the intensity of cellular respiration and showed that both the level of oxygen uptake and the ADP dissociation constant markedly decreased in comparison with values in the control mice [133].

Since, in the early stages of gravity disuse, the relative desmin content in m. soleus rat fibres decreases [8, 122], this suggests that the speed of cellular respiration will decrease. These findings showed that cellular respiration decreased after three days of gravity disuse, achieved its minimum after seven days, and increased to the control level on the fourteenth day of simulated disuse [134]. A three-day reloading period leads to some decrease in the cellular respiration, but recovery of the respiration parameters up to the control level was observed after seven days of readaptation [135]. After two days of immobilisation of rat hindlimbs, a decrease in the respiration speed (by 37%) was determined in the subsarcolemmal mitochondria extracted from the m. soleus [136]. The ADP-stimulating speed of cellular respiration of the vastus lateralis muscle of two monkeys, under gravity-free conditions for 15 days, decreased by 28% and 32% [137]. Meanwhile, according to Bigard et al. [138], a disuse, for three weeks, did not cause definitive changes in the oxygen uptake of skinned soleus fibres.

During work on cardiomyocytes, Saks et al. [139] showed that the intensity of oxygen uptake depends on the state of the cytoskeleton. Our findings showed that the basal speed of cellular respiration of rat cardiomyocytes did not, in practical terms, change during antiorthostatic disuse; it did increase, but insignificantly, during the first day. Meanwhile on adding glutamate and malate to the medium, the respiration speed and maximum respiration speed significantly increased after just one day, and remained high for the whole period of disuse up to the fourteenth day. Thus, after a 3-day-reloading after 14 days of antiorthostatic disuse, all indicated parameters significantly decreased in comparison to the control level [6]. However, Bigard et al. [138] did not find any changes in the intensity of cellular respiration in cardiomyocytes after three weeks of antiorthostatic suspension. The discrepancy between our results and those of Bigard et al. [138] can be accounted for by different durations of the display. Although the primary increase in maximum respiration speed and respiration speed on the exogenic substrates shows that the numbers of mitochondria, and/or concentrations of the respiration chain complexes in them, can increase, this does not cause the increase in the basal respiration speed in the context of unchanged quantities of endogenic substrates. The increase in the relative content of desmin in rat cardiomyocytes under conditions of antiorthostatic disuse [6], which is necessary for the definition of mitochondria localisation and regulation of the permeability of their membranes, in combination with data on the increase of other oxidative enzyme and mitochondrial creatin kinase contents, prove this hypothesis [140].

## 7. Conclusion and Perspectives

The gravitational field is the most constantly acting factor across the whole evolutionary development of all living organisms on Earth. Therefore, it is logical to suggest that mechanosensory mechanisms explained acts of the primary receptions of the mechanical force could be universal for the different cells. Indeed, they can be connected with the most ancient of cell structures, the membrane with the cortical cytoskeleton.

The data presented in the literature prove that cell mechanical properties, particularly stiffness and the Young's modulus, are defined mostly by the state of the actin cytoskeleton and not by the microtubule system. However, the means of forming a state of tension through the system of thin filaments is not yet clear enough. There are at least two possible ways of realising this function. The first being that actin forms a reticulum which is stiff enough to play the role of a skeleton. The second is through actin-myosin interactions, which are not exclusive to just the muscle cells. The findings of Martens and Radmacher [10] on human fibroblasts showed that cell stiffness can be explained by the tension created by myosin transferring along the actin filaments.

There is no doubt that external surroundings influence cell mechanical properties, most likely by means of reorganisation of the actions of the cytoskeleton. The data on cells cultured on different substrates proves this suggestion. Both the topography of the substrate and its stiffness are of great importance. The data, obtained with the use of agents that damage the actin network meanwhile, show a leading role for the thin filament system in forming the cell structure and its rapid responses to changes in external conditions.

Additionally, it was shown that different pathologic processes, especially malignant transformation of cells, can cause changes in the mechanical properties that tend to increase cell stiffness. Evidently, this is also connected with the impaired regulation of the actin cytoskeleton state that takes place during the increase in cell proliferation speed. On the other hand, aging of the organism causes decreases in the stiffness of some cells that can be also connected with a change in cytoskeleton state.

A change in the mechanical conditions also causes a change in cell stiffness, in particular in skeletal muscle fibres and cardiomyocytes. The decrease in the external mechanical force causes a decrease in the stiffness, and an increase, which in itself is correlated to the dynamics of nonmuscle actin isoform (beta- and gamma-) contents in the membrane fraction of muscle cells, is connected with differently directed changes in alpha-actinin-1 and alpha-actinin-4 contents in the cytoplasmic and membrane protein fractions.

The increase in an external mechanical force, naturally, should cause an increase in a cell's ability to resist it, that is, an increase in cell stiffness and development of the cytoskeleton. A decrease in the force does not require a developed cytoskeleton, and as a result the cell stiffness should decrease. Thus, it can be supposed that regulation of the actin cytoskeleton state plays the leading role in the change of speed of cell division, development of pathologic processes, and responses to the change in cellular mechanical conditions (hypo- and hyper-gravity), causing subsequent alterations in the cell's mechanical properties.

**Figure 2:**
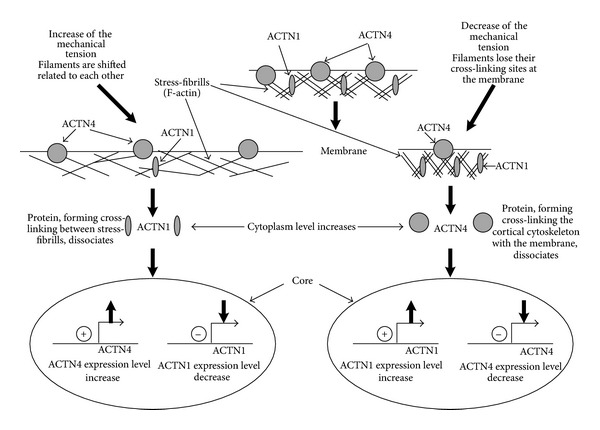
Hypothetical mechanism for earlier cellular responses to changes in mechanical conditions. The principal difference between stretch and compression is characterized by the dissociation of different molecules from the cortical cytoskeleton, for example, alpha-actinin-1 at stretching and alpha-actinin-4 at compression. Under cell stretch, there are cortical cytoskeleton deformations and subsequent shifts in actin filaments relative to each other in the stress fibres. This increases the probability of dissociation of the proteins that connect actin filaments, for example, alpha-actinin-1. Under cell compression, this happens predominantly via membrane deformation so that the conformation of the alpha-actinin-4 binding sites (e.g., due to cholesterol raft convergence) can change. This will lead to a release of proteins which connect with the membrane, for example, alpha-actinin-4. The release of alpha-actinin-4 causes the activation of the expression of the alpha-actinin-1 gene and repression of own expression. This occurs similarly for alpha-actinin-1. The release of different proteins causes activation of different pathways and formation of the response to the increase or decrease in mechanical load. The proposed mechanism is only hypothetical and therefore needs to be checked experimentally.

**Figure 3:**
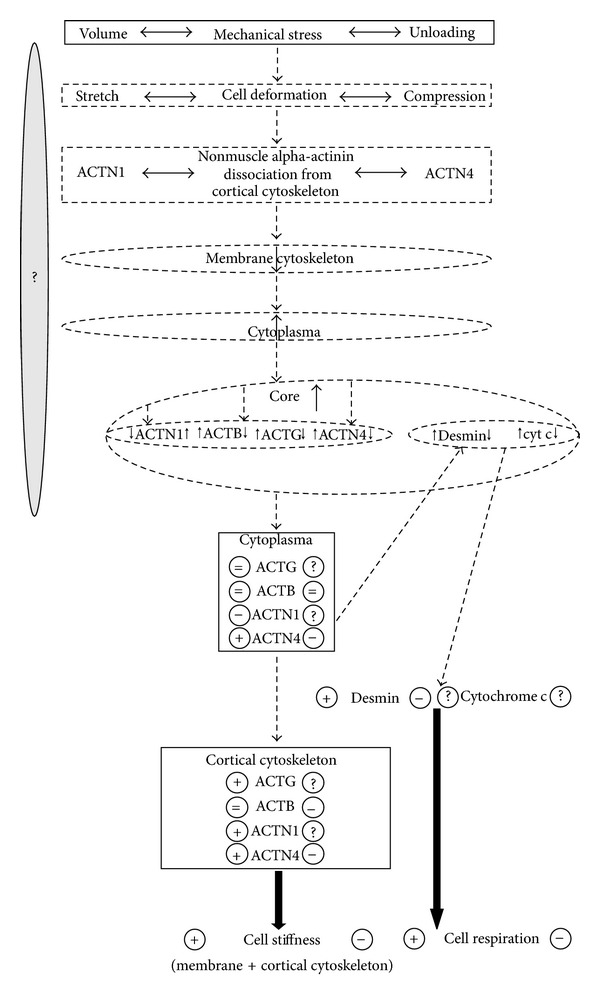
Hypothetical mechanism for the development of adaptive responses of skeletal muscle fibres and cardiomyocytes to the change in mechanical conditions. An increase in the external mechanical load on the cardiomyocytes in early stages of antiorthostatic disuse in rats should naturally cause an increase in the cell's ability to resist it, that is, an increase in cell stiffness and development of the cytoskeleton, in addition to an intensification of the cell respiration. Decrease of the load on the rat skeletal muscle fibres during antiorthostatic disuse does not require the development of the cortical cytoskeleton, and as a result the cell's stiffness should decrease. Hypothetical links are shown by dashed arrowheads and contours. −/+: decrease/increase in protein content, from the left/to right—for load/disuse.

Thus, by summarising the data, we can suggest (Figures 2 and 3) that a decrease in mechanical load causes the decrease in skeletal muscles fibre stiffness, likely due to the decrease in nonmuscle actin isoforms and alpha-actinin content in the membrane protein fraction, and also the decrease in cell respiration intensity that takes place in the skeletal muscles. At the increase of mechanical load on cells, in particular on the cardiomyocytes, during antiorthostatic disuse in rats, causes an intensification of the processes of cellular respiration, an increase of nonmuscle actin and alpha-actinin content in the membrane protein fraction, and an increase in cell stiffness also. Therefore, there are differently directed changes in the alpha-actinin-1 and alpha-actinin-4 content in the cytoplasmic protein fractions.

The results discussed here suggest several approaches to the regulation of cell mechanosensitivity and present a range of possibilities for prevention of the negative effects of microgravity and correction of negative changes connected with hypogravity syndrome. For example, the use of gene constructs or any preparations which provide stabilisation of the cortical cytoskeleton could prevent the negative effects of exposure to a zero-gravity environment. However, the problem of perception of mechanical stimuli by different cells still requires further study.
